# The impact of *SETBP1* mutations in neurological diseases and cancer

**DOI:** 10.1111/gtc.13057

**Published:** 2023-07-25

**Authors:** Naoki Kohyanagi, Takashi Ohama

**Affiliations:** ^1^ Laboratory of Veterinary Pharmacology, Joint Faculty of Veterinary Medicine Yamaguchi University Yamaguchi Japan

**Keywords:** cancer, neurological disease, PP2A, SET, SETBP1

## Abstract

SE translocation (SET) is a cancer‐promoting factor whose expression is upregulated in many cancers. High SET expression positively correlates with a poor cancer prognosis. SETBP1 (SET‐binding protein 1/SEB/MRD29), identified as SET‐binding protein, is the causative gene of Schinzel–Giedion syndrome, which is characterized by severe intellectual disability and a distorted facial appearance. Mutations in these genetic regions are also observed in some blood cancers, such as myelodysplastic syndromes, and are associated with a poor prognosis. However, the physiological role of SETBP1 and the molecular mechanisms by which the mutations lead to disease progression have not yet been fully elucidated. In this review, we will describe the current epidemiological data on SETBP1 mutations and shed light on the current knowledge about the SET‐dependent and ‐independent functions of SETBP1.

## Graphical abstract

SE translocation (SET) is a cancer‐promoting factor whose expression is upregulated in many cancers. SETBP1 (SET binding protein 1) is the causative gene for Schinzel‐Giedion syndrome, and mutations in the same region are also observed in various blood cancers. In this review, we will describe the current epidemiological data on SETBP1 mutations and shed light on the current knowledge about the SET‐dependent and ‐independent functions of SETBP1.

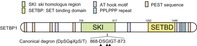

## 1 STRUCTURE OF SETBP1

**FIGURE 1:**
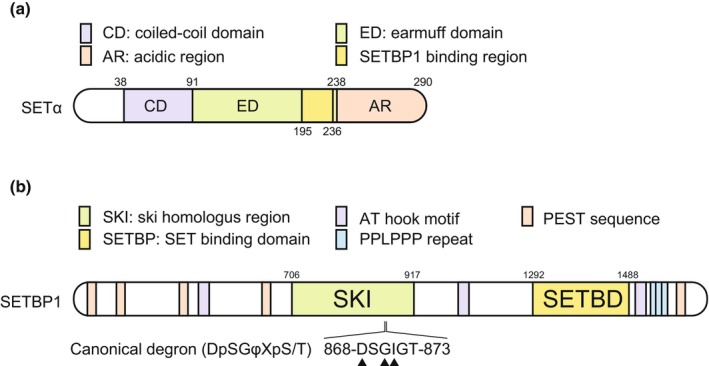
The structure of SET and SETBP1. (a) Schematic representation of SETα isoform. SETBP1 directly associates with 195–236 amino acid residues of SETα. (b) Schematic representation of SETBP1. The residues of canonical degron are located in the SKI region. Hotspot mutations observed in SGS patients are indicated by the arrowhead.

SET is an oncoprotein—also known as I2PP2A or TAF1—and was identified as a SET‐CAN fusion protein in acute non‐lymphocytic leukemia, produced by the deletion of chromosome 9 (Adachi et al., 1994). SET is a multifunctional protein that acts as an inhibitor of protein phosphatase 2A (PP2A), a major serine/threonine protein phosphatase. Additionally, it also acts as an epigenetic modulator through its function as a histone chaperone and as an inhibitor of the histone acetyl‐transferase complex (INHAT) (Dacol et al., 2021). Furthermore, it is a tumor‐promoting factor, and increased SET expression has been reported in various types of cancers. This protein is composed of a coiled‐coil domain (CD) responsible for dimerization, an earmuff domain (ED), and an acidic region (AR) (Figure 1a) (Bayarkhangai et al., 2018).

SETBP1 was identified as a SET‐binding protein using yeast two‐hybrid screening (Minakuchi et al., 2001). There are two transcriptional variants of SETBP1 that result in the synthesis of two isoforms, namely SETBP1a and SETBP1b. They are, approximately, 170 and 20 kDa, respectively. Exons 1–3 form a common region for both; SETBP1a is synthesized from six exons and SETBP1b from four exons. Previous studies have mainly focused on SETBP1a, and thus, the function of SETBP1b remains largely unknown (Coccaro et al., 2017). In this review, we will focus on SETBP1a and it will be referred to as SETBP1 hereafter.

SETBP1 is widely conserved ranging from insects to mammals; however, it is absent in yeast and nematodes. Human SETBP1 has a homologous region of the cancer‐promoting gene ski (SKI homologous region), six PEST sequences, three consecutive PPLPPP repeats, and three AT‐hook motifs (Figure 1b). Asp1292‐Lys1488 of SETBP1 is the SET‐binding domain (SETBD) that binds to Gln182‐Pro223 in the ED of SET (Minakuchi et al., 2001; Piazza et al., 2013).

**FIGURE 2:**
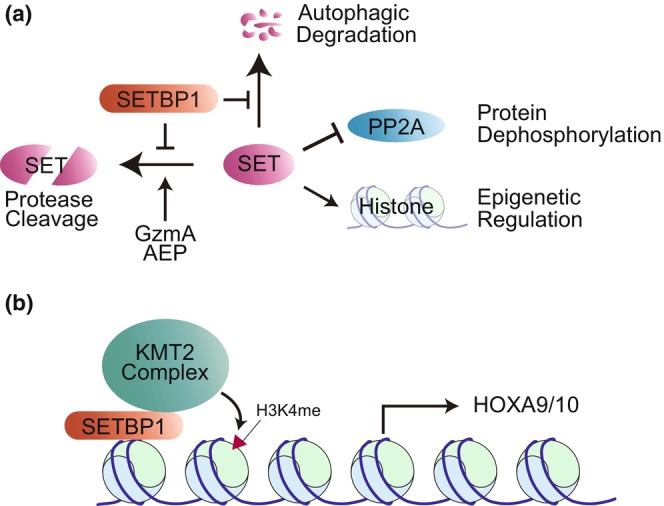
SET‐dependent and ‐independent functions of SETBP1. (a) SETBP1 stabilizes SET protein by blocking SET cleavage by proteases, such as granzyme A (GzmA) and asparaginyl endopeptidase (AEP), as well as autophagic degradation. As SET directly associates with PP2A and inhibits its phosphatase activity, SET accumulation by SETBP1 affects global phosphorylation signals. SET also functions as a histone chaperone. Therefore, SETBP1 may be involved in epigenetic regulation. (b) SETBP1 associates with AT‐rich promoter region and recruits the KMT2A complex. This causes methylation of Lys4 of histone H3 (H3K4me) and induces *HOXA9/10* expression.

SETBP1 proteins are ubiquitously expressed in the body and are most notably localized in the cell nuclei. The most well‐established function of SETBP1 is the suppression of PP2A activity via the stabilization of the SET proteins. SETBP1 binds directly to SET and accumulation of SETBP1 indirectly suppresses PP2A activity by protecting SET from cleavage by proteases and degradation via autophagy (Figure 2a) (Basurto‐Islas et al., 2013; Cristóbal et al., 2010; Fan et al., 2003; Kohyanagi et al., 2022; Piazza et al., 2013). SET also functions as an epigenetic modulator; although, this functional aspect has not received much attention with regard to SETBP1. This suggests that SETBP1 may also be involved in epigenetic regulation.

In contrast, the SET‐independent functions of SETBP1 have been thoroughly investigated in previous studies. In mouse myeloid progenitor cells, SETBP1 induces the expression of the homeobox proteins HOXA9 and HOXA10 and promotes their self‐renewal (Oakley et al., 2012; Sakaguchi et al., 2013). Additionally, SETBP1 binds to the AT‐rich promoter region of genomic DNA via its AT‐hook motif and recruits the KMT2A (MLL1) complex. This causes methylation of Lys4 in histone H3 and induces target gene expression (Figure 2b) (Nguyen et al., 2022; Piazza et al., 2018).

## 2 SETBP1 MUTATIONS IN NEUROLOGICAL DISEASES

Schinzel–Giedion syndrome (SGS), first described in 1978, is an autosomal dominant disorder characterized by severe intellectual disability, distorted facial features, skeletal abnormalities, and various congenital malformations of multiple organs (Schinzel & Giedion, 1978). It is a fatal disease that kills about half of the patients by 2 years and most by 10 years (Touge et al., 2001). De novo heterozygous mutations in the *SETBP1* gene were identified in unrelated SGS patients with SGS. Furthermore, Sanger sequencing identified similar mutations in eight out of the nine SGS patients that were evaluated (Hoischen et al., 2010; Suphapeetiporn et al., 2011). These mutations were located within the SKI homologous region and were not found in parents of the patients or healthy individuals. Moreover, p.G870S was the most frequently found mutation with an incidence of approximately 27% (7/26 patients) (Leone et al., 2020). These findings suggest that *SETBP1* is the major causative gene of this disease. Recently, SETBP1 mutations have also been observed in psychiatric disorders such as developmental epileptic encephalopathy, eating disorders, and autism (Table 1) (Alsubaie et al., 2020; Hu et al., 2022; Martínez‐Magaña et al., 2022).

**TABLE 1:** *SETBP1* mutation and diseases.

Variants	Biochemical functions	Diseases	References
p.G15R	LoF	SGS	(Coe et al., 2014)
p.R143V	LoF	SGS	(Coe et al., 2014)
p.E244Dfs8	ND	CML	(Romzova et al., 2021)
p.N272D	ND	GCT	(H. Wang et al., 2022)
p.L411G	LoF	SGS	(Coe et al., 2014)
p.W532	LoF	SGS	(Coe et al., 2014)
p.R589*	LoF	ID	(Leonardi et al., 2020)
p.K592	LoF	SGS	(Coe et al., 2014)
p.R625	LoF	SGS	(Coe et al., 2014)
p.R626	LoF	SGS	(Coe et al., 2014)
p.R627C	ND	AML	(Li et al., 2019)
p.E734Afs19	LoF	ID	(Leonardi et al., 2020)
p.Q766R	ND	MDS	(Polprasert et al., 2022)
p.I822Y	LoF	SGS	(Coe et al., 2014)
p.E858K	ND	DEE, ccRCC, aCML	(Leonardi et al., 2020; Piazza et al., 2013)
p.E862K	ND	SGS, EP	(Acuna‐Hidalgo et al., 2017; Balciuniene et al., 2019)
p.S867G	ND	MPN	(Eder‐Azanza et al., 2019)
p.S867R	ND	SGS	(Acuna‐Hidalgo et al., 2017; Carvalho et al., 2015)
p.D868A	ND	SGS	(Hoischen et al., 2010)
p.D868N	PS	SGS, aCML, sAML, RAEB, CMML1, CMML2, CNL, JMML, PT, MDS	(Carvalho et al., 2015; Cui et al., 2014; Elliott et al., 2015; Gao et al., 2020; Hirao et al., 2022; Hoischen et al., 2010; Kwon et al., 2022; Li et al., 2019; Makishima et al., 2013; Montalban‐Bravo et al., 2021; Mori et al., 2022; Piazza et al., 2013; Polprasert et al., 2022; Volk et al., 2015; Wakamatsu et al., 2021; Yin et al., 2019)
p.D868Y	ND	CMML2	(Makishima et al., 2013)
p.S869C	ND	SGS	(Landim et al., 2015)
p.S869G	ND	aCML	(Piazza et al., 2013)
p.S869N	ND	SGS	(Acuna‐Hidalgo et al., 2017)
p.S869R	ND	SGS	(Acuna‐Hidalgo et al., 2017)
p.G870C	ND	SGS	(López‐González et al., 2015)
p.G870D	ND	SGS, CNL, CL	(Acuna‐Hidalgo et al., 2017; Elliott et al., 2015; Hoischen et al., 2010; Mori et al., 2022)
p.G870N	ND	CNL	(Cui et al., 2014)
p.G870S	PS	SGS, aCML, CMML, CNL, sAML, JMML PMF, PNH, MDS	(Fontana et al., 2020; Herenger et al., 2015; Hirao et al., 2022; Hoischen et al., 2010; Kim et al., 2021; Ko et al., 2013; Kwon et al., 2022; Leone et al., 2020; Makishima et al., 2013; Montalban‐Bravo et al., 2021; Park et al., 2021; Piazza et al., 2013; Polprasert et al., 2022; Qian et al., 2021; Suphapeetiporn et al., 2011; Yun et al., 2020)
p.I871S	ND	SGS	(Sullivan et al., 2020; Takeuchi et al., 2015)
p.I871T	PS	SGS, aCML, CNL, sAML, FA	(Acuna‐Hidalgo et al., 2017; Hirao et al., 2022; Hoischen et al., 2010; Lestner et al., 2012; Makishima et al., 2013; Miyake et al., 2015; Pergande et al., 2020; Piazza et al., 2013; Yin et al., 2019)
p.T873I	ND	SGS	(Acuna‐Hidalgo et al., 2017)
p.D874N	ND	CNL	(Cui et al., 2014)
p.D880E	ND	sAML	(Makishima et al., 2013)
p.D880N	ND	CMML1	(Makishima et al., 2013)
p.S893Y	ND	JMML	(Kim et al., 2021)
p.R972W	ND	BIA‐ALCL	(Fiore et al., 2020)
p.S1011	LoF	SGS	(Coe et al., 2014)
p.S1076L	ND	ID, IAC	(Leonardi et al., 2020)
p.T1078M	ND	JMML	(Kim et al., 2021)
p.H1116R	ND	NSID	(Taşkıran et al., 2021)
p.A1193T	ND	AML	(Li et al., 2019)
p.V1377L	ND	BC	(Glentis et al., 2019)
p.E1466D	ND	AML	(Li et al., 2019)
p.P1563L	ND	AML	(Li et al., 2019)
Fusion with *NOTCH3*	ND	MGM	(Khan et al., 2020)
Fusion with *NPMI*	ND	AML	(Martelli et al., 2021)

Abbreviations: aCML, atypical chronic myelogenous leukemia; BC, breast cancer; BIA‐ALCL, breast implant‐associated anaplastic large cell lymphoma; ccRCC, clear cell renal carcinoma; CL, chronic leukocytosis; CMML1/2, chronic myelomonocytic leukemia 1/2; CNL, chronic neutrophilic leukemia; DEE, Developmental and epileptic encephalopathies; EP, epilepsy; FA, fetal akinesia; GCT, germ cell tumor; IAC, intestinal adenocarcinoma; ID, intellectual disability; ITAC, intestinal‐type adenocarcinoma; JMML, juvenile myelomonocytic leukemia; LoF, loss of function; MDS, myelodysplastic syndromes; MGM, meningioma; MPN, myeloproliferative neoplasms; ND, not determined; NSID, non‐specific intellectual disability; PMF, primary myelofibrosis; PNH, paroxysmal nocturnal hematuria; PS, protein stabilization; PT, persistent thrombocytopenia; RAEB, refractory anemia with ringed sideroblasts; sAML, secondary acute myeloid leukemia; SGS, Schinzel–Giedion syndrome.

*SETBP1* mutations are involved in early‐stage neuronal degeneration and lead to SGS pathogenesis. It is known that the SKI homology region is a binding site for the ubiquitin ligase β‐TrCP1; these mutations reduce SETBP1 binding to β‐TrCP1. Furthermore, they inhibit SETBP1 degradation via the ubiquitin‐proteasome system (Piazza et al., 2013). Recently, iPS cells were established from SGS patients and used to demonstrate that SETBP1 mutations in neural progenitor cells accumulate DNA damage by upregulating SET expression and inhibiting p53 transcriptional activity (Banfi et al., 2021). SET‐mediated p53 inhibition is mediated by SET‐inhibitory effect on histone acetylation. SET is a component of the INHAT complex that masks lysine residues on histones and other proteins; this protects them from acetylation by p300/CBP and PCAF. It has also been reported to bind to non‐acetylated lysine residues at the C‐terminus of p53 and inhibit histone H3 acetylation. This results in the repression of p53 target gene transcription (Kim et al., 2012; Wang et al., 2016).

Furthermore, the loss‐of‐function mutations in SETBP1 have also been reported to be associated with intellectual disability, expressive language disorders, childhood aphasia, autism spectrum disorders, neurodevelopmental disorders, and developmental epileptic encephalopathy (Antonyan & Ernst, 2022; Coe et al., 2014; Filges et al., 2011; Jansen et al., 2021; Leonardi et al., 2020; Morgan et al., 2021; Rakhlin et al., 2020; Wong et al., 2022). These symptoms are also observed in SGS but are more severe. Therefore, appropriate SET regulation by SETBP1 may be important for normal neural development.

## 3 SETBP1 MUTATIONS IN CANCER

Elevated SETBP1 expression has been identified in many hematologic cancers. These include acute myeloid leukemia (AML), chronic myelomonocytic leukemia (CMML), myelodysplastic syndrome (MDS), and solid tumors, such as bladder cancer. Its expression is also associated with poor prognosis (Cristóbal et al., 2010; Eisfeld et al., 2020; Elena et al., 2016; Hwang et al., 2019; Jiang et al., 2020; Li et al., 2021; Lucas et al., 2018; Martín et al., 2020; Robinson et al., 2020; Tang et al., 2022). The major cause of elevated SETBP1 expression is mutations in and around the SKI region; however, there are also known cases that occur in the absence of SETBP1 mutations (discussed later).

*SETBP1* mutations in cancer were first reported as *NUP98‐SETBP1* fusion genes in patients with T‐cell acute lymphoblastic leukemia (Panagopoulos et al., 2007). Later, it was discovered that mutations in the SKI homologous region found in SGS patients were also present in cancer. Exome sequencing performed on atypical chronic myeloid leukemia (aCML) identified *SETBP1* mutations in 19 of 78 cases, 92% of which were located in the SKI homologous region (Makishima et al., 2013). aCML patients with *SETBP1* mutations showed signs of acute transformation in blood tests and had a shorter overall survival rate than the healthy controls (Meggendorfer et al., 2013). Specific mutations in Asp868, Ser869, Gly870, Ile871, and Asp880 within the SKI homology region have also been reported in approximately 15% of CMML, a type of MDS, and 17% of secondary AML that progressed from MDS (Han et al., 2022; Makishima et al., 2013; Nie et al., 2022). These were also associated with a shorter overall survival rate. These SETBP1 mutations have rarely been identified in non‐secondary acute myeloid leukemia (Badar et al., 2020; Makishima et al., 2013). A cohort study of 100 MDS patients showed that the SETBP1 mutation rate is higher in males; their prognosis is worse than that of females (Karantanos et al., 2021). Bone marrow fibrosis (BMF) is a known poor prognostic factor in MDS. *SETBP1* mutations were identified at a higher rate in the BMF grade 3 group than in the grade 0–2 group (Melody et al., 2020). These findings suggest that mutations in the SKI region of SETBP1 are a potential cause for the transformation of MDS into leukemia (Makishima et al., 2013). *SETBP1* mutations have also been identified in solid tumors including pancreatic neuroendocrine tumors, breast cancer, non‐small cell lung cancer, and gastric cancer (Ban et al., 2022; Coudray et al., 2018; Glentis et al., 2019; Zhang et al., 2019).

The co‐existence of certain genetic mutations along with *SETBP1* mutations leads to a worse prognosis. Of the 368 MDS patients studied, 64 (17.4%) possessed mutations in the *ASXL1* gene, encoding ASXL transcriptional regulator 1 (Inoue et al., 2015). Furthermore, 9.4% of the 64 patients with *ASXL1* mutations also had *SETBP1* mutations. In contrast, the *SETBP1* mutation rate in *ASXL1* mutation‐negative patients was only 0.7%. MDS with *ASXL1* and *SETBP1* co‐mutations had a higher rate of conversion to AML and a shorter overall survival rate compared to that of *ASXL* mutations alone (Inoue et al., 2015). In CMML, 36% (9/25) of patients with *ASXL1* mutations carried *SETBP1* mutations, while 81.8% (9/11) of patients with *SETBP1* mutations were positive for *ASXL1* mutations (Mason et al., 2016). These data suggest that *ASXL1* mutation is followed by *SETBP1* mutations, thus leading to disease progression. Mutation analysis of various myeloproliferative neoplasms classes also showed the prevalence of *ASXL1* and *SETBP1* co‐mutations in aCML (Palomo et al., 2020). Accumulating evidence has shown that *ASXL1* mutations inhibit cancer cell differentiation and that *SETBP1* mutations promote cell proliferation, thus leading to AML progression (Inoue et al., 2015; Makishima et al., 2013; Saika et al., 2018). It has also been reported that the presence of *ASXL1* and *SETBP1* mutations reduces histone H3 and H4 acetylation levels near the TGFβ target gene promoter region and inactivates the TGFβ pathway, thus inhibiting AML progression (Inoue et al., 2015; Makishima et al., 2013; Saika et al., 2018).

Association of *SETBP1* with mutations other than *ASXL1* has also been reported. *SETBP1* mutations have been shown to be a poor prognostic factor in chronic neutrophilic leukemia (CNL) which is a rare myeloproliferative leukemia (Gao et al., 2022). Patients with colony‐stimulating factor 3 receptor (*CSFR3*) mutations, which account for more than half of the CNL patients, have a high frequency of *SETBP1* mutations. The concomitant occurrence of these mutations exacerbates CNL by upregulating the expression of c‐Myc and its target genes (Anil et al., 2021; Carratt et al., 2022; Qian et al., 2021). Approximately, 85% of juvenile myelomonocytic leukemia (JMML) patients have *JAK3* mutations. The SETBP1 p.G870N mutation co‐exists frequently with JAK3 mutations and is associated with a poor prognosis (Wakamatsu et al., 2021). In JMML, SETBP1 enhances *NRAS* gene expression, causing the activation of mitogen‐activated protein kinase signaling and repression of differentiation (Carratt et al., 2021). MDS with mutations in *U2FA1* gene, which encodes U2 small nuclear RNA auxiliary factor 1, is associated with a poor prognosis and is prone to mutations in *SETBP1* (H. Wang et al., 2020). The prognosis of MDS and other hematologic cancers patients with isochromosome 17q (i(17q)), a monosomy of the short arm and trisomy of the long arm of chromosome 17, is poor. *SETBP1* mutation rates are higher in these cancers, and they frequently co‐exist with mutations in the serine and arginine‐rich splicing factor 2 (*SRSF2*) gene that regulates RNA splicing (Kanagal‐Shamanna et al., 2022).

## 4 INCREASED SETBP1 EXPRESSION IN CANCER

**FIGURE 3:**
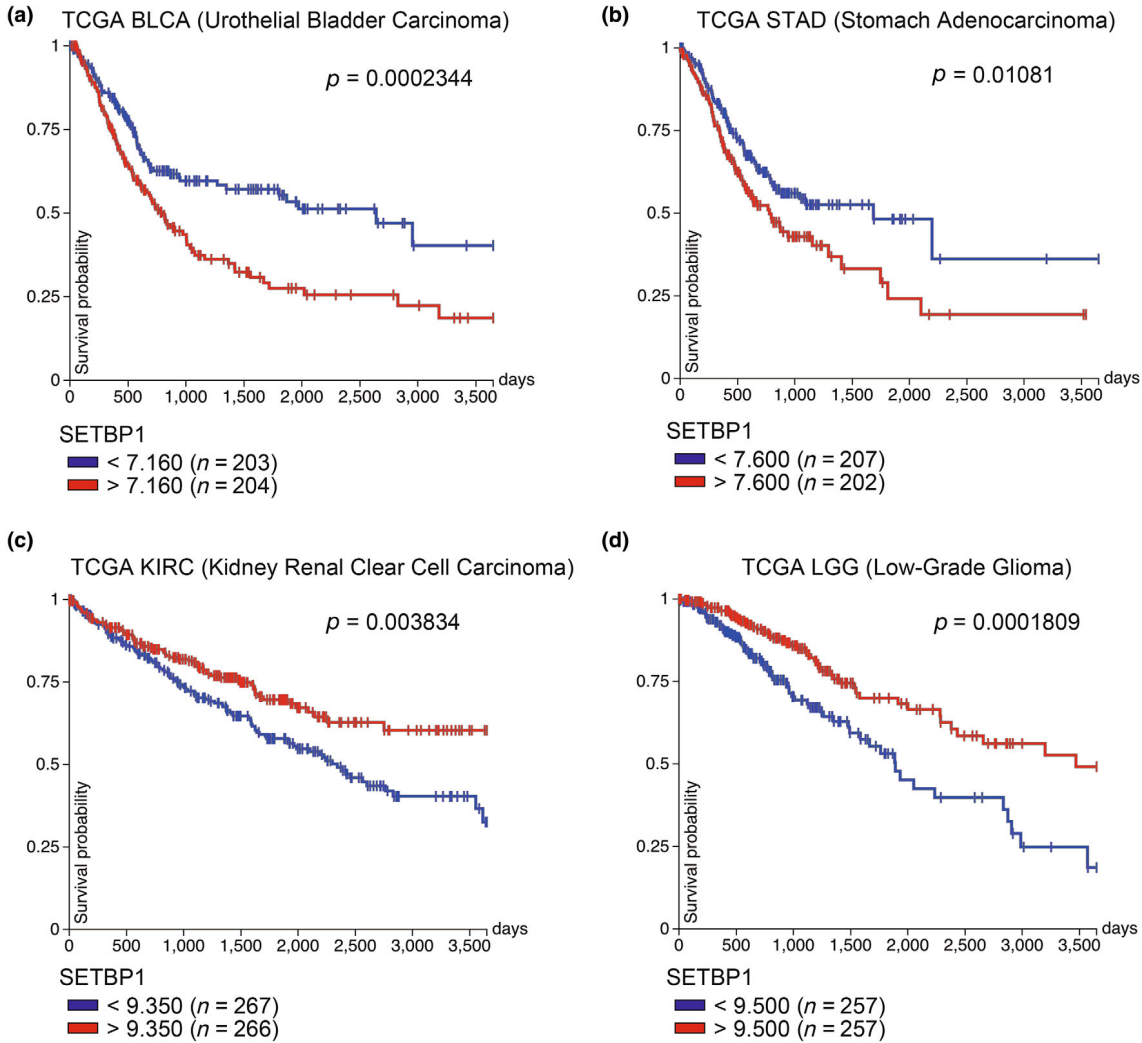
SETBP1 expression and prognosis of cancer patients. Kaplan–Meier curves for SETBP1 expression and 10‐year survival. TCGA dataset for (a) urothelial bladder carcinoma (BLCA), (b) stomach adenocarcinoma (STAD), (c) kidney renal clear cell carcinoma (KIRC), and (d) low‐grade glioma (LGG).

Mutations in the SKI region contribute to cancer malignancy by stabilizing the SETBP1 protein; on the other hand, increased SETBP1 protein not caused by mutations has also been reported. In AML, the t(12; 18)(p13; q12) translocation causes increased expression of SETBP1; the translocation is located near its cleavage point (Cristóbal et al., 2010). It is associated with a shorter overall survival rate. Owing to this translocation, elevated SETBP1 expression associated with decreased miR‐4319 expression has also been observed in patients who progressed from primary myelofibrosis to AML (Albano et al., 2012, p. 1). Additionally, SETBP1 expression was higher in younger patients with an adverse karyotype of AML (Lucas et al., 2018). According to The Cancer Genome Atlas Program (TCGA) database, patients with high SETBP1 expression in urothelial bladder carcinoma and stomach adenocarcinoma have a poor prognosis (Figure 3a,b).

Internal tandem duplication caused by mutations in the *FLT3* gene (*FLT3‐ITD*) leads to the constitutive activation of FMS‐like tyrosine kinase 3 (FLT3). FLT3‐ITD promotes AML development, in concert with oncogenic mutations and chimeric fusion genes, which are referred to as the “class‐defining” mutations. However, approximately 20% of FLT3‐ITD‐positive AML cases do not possess these class‐defining mutations. In some of these cancers, elevated SETBP1 expression appears to cooperate with FLT3‐ITD to trigger AML development (Pacharne et al., 2021).

On the other hand, lower SETBP1 expression also correlates with a poor prognosis. According to TCGA database, patients with low SETBP1 expression in kidney renal clear cell carcinoma and low‐grade glioma had a poor prognosis (Figure 3c, d). In non‐small cell lung cancers, decreased SETBP1 expression has been reported to cause epithelial‐mesenchymal transition and cell proliferation via ERK signaling (Li et al., 2020). It has also been reported that SETBP1 expression decreases in the stroma as breast cancer progresses from grades I to III (Uddin & Wang, 2022).

**FIGURE 4:**
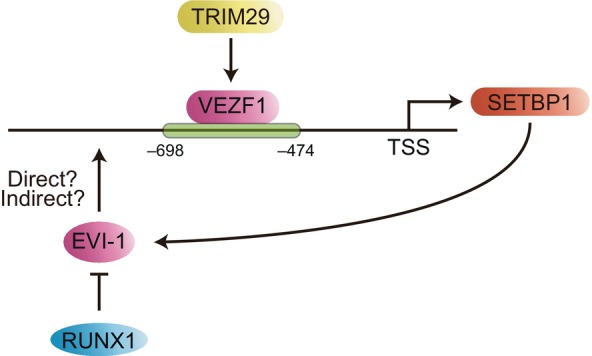
Regulation of SETBP1 transcription. Tripartite motif‐containing 29 (TRIM29) promotes the expression of the transcriptional factor VEZF1. VEZF directly binds to the *SETBP1* promoter region and induces SETBP1 transcription. Transcriptional factor EVI‐1 directly or indirectly induces SETBP1 expression. The expression of EVI‐1 is positively and negatively regulated by SETBP1 and RUNX1, respectively.

The molecular mechanisms that regulate SETBP1 transcription are not fully understood; however, some have recently been elucidated. Vascular endothelial zinc finger 1 (VEZF1/ZNF161), a transcription factor with a C2H2‐type zinc finger motif, binds to the *SETBP1* promoter region and upregulates its expression in ovarian cancer (Figure 4) (Qiao et al., 2022). This is caused by the transcription of VEZF1 that is induced by a tripartite motif containing 29 (TRIM29). TRIM29 is upregulated in pancreatic, gastric, lung, bladder, and ovarian cancers; its expression levels are known to correlate with tumor size and grade.

Ecotropic virus integration site 1 protein homolog (EVI‐1) is an essential transcriptional factor for the proliferation of hematopoietic stem and leukemic cells. It is not clear whether EVI‐1 directly binds to the promoter region of SETBP1; however, SETBP1 expression is decreased by EVI‐1 deficiency (Goyama et al., 2008). *RUNX1* is frequently mutated in human leukemia patients, and the mutated protein is involved in leukemogenesis through a dominant‐negative effect on the normal RUNX1. Analysis of the TCGA database revealed that SETBP1 expression was elevated in AML cells with *RUNX1* mutations (Pacharne et al., 2021). EVI‐1 expression was also elevated in AML cells with *RUNX1* mutations, suggesting that *RUNX1* mutations may upregulate SETBP1 expression via EVI‐1 (Watanabe‐Okochi et al., 2008, p. 1). Furthermore, SETBP1 binds to the promoter region of *EVI1* and promotes EVI‐1 expression (Piazza et al., 2018). Both EVI1 and SETBP1 upregulation promote bone marrow hematopoiesis (Ott et al., 2006). Cases of CNL with simultaneous EVI‐1 upregulation and SETBP1 mutations have also been reported (Altangerel et al., 2015). These observations suggest a dynamic interaction between EVI‐1 and SETBP1 in hematopoietic stem cells and leukemia cells.

Although the detailed molecular mechanisms are unclear, increased cell density promotes *SETBP1* transcription in adherent cells (Kohyanagi et al., 2022). Elevated SETBP1 expression at high cell densities is a common phenomenon observed in many normal and cancer cell lines. Increased cell density causes acidification of the microenvironment, hypoxia, low nutritional status, and increased physical pressure. AMP‐activated protein kinase (AMPK) is a metabolic master switch that maintains cellular energy homeostasis. According to the Human Gene Database GeneCards, several AMPK‐downstream transcription factors, such as MEF2 and FOXO4, bind to the promoter region of *SETBP1*. Therefore, changes in nutritional status associated with increased cell density and decreased blood flow in cancer tissues may contribute to increased SETBP1 expression.

## 5 CONCLUSION AND PERSPECTIVE

Here, we described the impact of *SETBP1* mutations in neurological diseases and cancer, in addition to their molecular mechanisms. Although numerous SETBP1 mutations have been reported in both diseases, the knowledge of why these mutations cause this pathology is limited. The function of SETBP1 is largely explained by the stabilization of SET and a concomitant decrease in PP2A activity. However, SET also functions as a histone modulator. SETBP1 also appears to have SET‐independent functions. Therefore, future research should not be limited only to the SETBP1/SET/PP2A axis.

While SETBP1 mutations have been identified in the SKI homology region in both SGS and cancer, differences exist between the two diseases. Mutations in Ile871 are more frequent in SGS than in cancer, and they have a relatively weak effect on SETBP1 protein cellular accumulation (Acuna‐Hidalgo et al., 2017). The Asp868 mutation, however, is more frequent in cancer and causes more pronounced SETBP1 accumulation. Additionally, SGS patients with Asp868 mutations are more susceptible to cancer than those with Ile871 mutations. These observations indicate that the threshold for causing cancer is higher than SGS. The phenotypic differences arising from the mutations within each residue require further analyses.

## AUTHOR CONTRIBUTIONS

Takashi Ohama and Naoki Kohyanagi wrote this article.

## FUNDING INFORMATION

This work was partially supported by JSPS KAKENHI (grant numbers 20H03151 and 22J23055).

## CONFLICT OF INTEREST STATEMENT

The authors declare that there is no conflict of interest regarding the publication of this paper.
